# Towards a Global Names Architecture: The future of indexing scientific names

**DOI:** 10.3897/zookeys.550.10009

**Published:** 2016-01-07

**Authors:** Richard L. Pyle

**Affiliations:** 1 Bernice Pauahi Bishop Museum, 1525 Bernice Street, Honolulu, HI 96817, USA

**Keywords:** Taxonomy, Carl Linnaeus, Charles Davies Sherborn, Biodiversity Data, Global Names Usage Bank, Global Names Index, ZooBank Biodiversity Library

## Abstract

For more than 250 years, the taxonomic enterprise has remained almost unchanged. Certainly, the tools of the trade have improved: months-long journeys aboard sailing ships have been reduced to hours aboard jet airplanes; advanced technology allows humans to access environments that were once utterly inaccessible; GPS has replaced crude maps; digital hi-resolution imagery provides far more accurate renderings of organisms that even the best commissioned artists of a century ago; and primitive candle-lit microscopes have been replaced by an array of technologies ranging from scanning electron microscopy to DNA sequencing. But the basic paradigm remains the same. Perhaps the most revolutionary change of all – which we are still in the midst of, and which has not yet been fully realized – is the means by which taxonomists manage and communicate the information of their trade. The rapid evolution in recent decades of computer database management software, and of information dissemination via the Internet, have both dramatically improved the potential for streamlining the entire taxonomic process. Unfortunately, the potential still largely exceeds the reality. The vast majority of taxonomic information is either not yet digitized, or digitized in a form that does not allow direct and easy access. Moreover, the information that is easily accessed in digital form is not yet seamlessly interconnected. In an effort to bring reality closer to potential, a loose affiliation of major taxonomic resources, including GBIF, the Encyclopedia of Life, NBII, Catalog of Life, ITIS, IPNI, ICZN, Index Fungorum, and many others have been crafting a “Global Names Architecture” (GNA). The intention of the GNA is not to replace any of the existing taxonomic data initiatives, but rather to serve as a dynamic index to interconnect them in a way that streamlines the entire taxonomic enterprise: from gathering specimens in the field, to publication of new taxa and related data.

“Global Names Architecture”

## Introduction

Although biological taxonomy is sometimes referred to as the “oldest profession” (Hedgpeth 1961, Chmielewski and Krayesky 2013), its current incarnation began with the start of modern nomenclature in the middle part of the eighteenth century (Linnaeus 1753, 1758). Throughout this time, the fundamental unit of taxonomy has been the “species”, the concept for which has eluded a clear consensus definition (e.g., Wheeler and Meier 2000). Linnaeus himself was a creationist, and therefore saw species as the work of God (Linnaeus 1736:18; translation from Wilkins 2009:41):

*Species tot sunt diversae quot diversas formas ab initio creavit infinitum Ens. [There are as many species as the Infinite Being produced diverse forms in the beginning.*]

This is not at all surprising, given that Darwin’s concept of evolution was not proposed until a century after the start of modern nomenclature (Darwin 1859). But even then, Darwin opted not to attempt a precise definition of “species”, writing (p. 40):

*Hence, in determining whether a form should be ranked as a species or a variety, the opinion of naturalists having sound judgment and wide experience seems the only guide to follow. We must, however, in many cases, decide by a majority of naturalists, for few well-marked and well-known varieties can be named which have not been ranked as species by at least some competent judges*.

This idea was reflected by the definition of species by Regan (1926: 75):

*A species is a community, or a number of related communities, whose distinctive morphological characters are, in the opinion of a competent systematist, sufficiently definite to entitle it, or them, to a specific name. [often paraphrased as, “a species is what a competent taxonomist says it is*”]

Many modern taxonomists have dismissed this definition as unscientific or too reliant on the notion of what “competent” means, and as a result, debates regarding a more precise and biologically meaningful definition of species have continued over the decades well into modern times (publications too numerous to cite, but see Wilkins 2009 for a review).

Regardless of its merit, acceptance, or adoption, a variant of this definition, effectively “a species is what a community of taxonomists says it is” is the *de-facto* species definition that has been applied since the time of Linnaeus. Taxonomists have asserted individual species circumscriptions over the course of centuries, and those circumscriptions that have met with approval by subsequent taxonomic communities have endured the test of time. In the modern context, while there are certainly species that are subject to ongoing debate, the vast majority of species have achieved some level of stability.

In stark contrast to the dynamic, ongoing, and seemingly endless debates about what a “species” is, the nomenclatural system used by taxonomists during the past two and a half centuries has been remarkably consistent, universal, and stable. The primary reason for this consistent and universal stability has to do with the Codes of scientific Nomenclature (e.g., ICZN 1999, Lapage et al. 1990, McNeill et al. 2012), which have enjoyed near-universal adoption for more than a century. A major reason for the contrast between “species” and scientific names is that the former are, and likely always will be subjective in their core nature; whereas the latter leverage the objectivity of the nomenclatural codes to reduce matters of opinion and dispute to a minimum. In effect, the Linnaean nomenclatural system represents a stable scaffolding against which which the ever-changing landscape of species can be reliably referenced.

It is the objective and largely stable nature of scientific names of organisms that makes them well-suited for large-scale indexing of the sort that Charles Davies Sherborn (1861–1942) dedicated his life to. Whereas the majority of the nearly 4,400 species circumscriptions described by Linnaeus in his 1758 *Systema Naturae* bear very little resemblance to the species boundaries asserted by modern biologists, most of the scientific names he established are not only available under the current Code, but are in current use (though often in combination with different generic names than what Linnaeus used). Even when historical scientific names have been synonymized by later workers, they remain available (when Code-compliant), and therefore potentially relevant centuries after their establishment. Although catalogs of species (e.g., Linnaeus 1758) may begin to lose their taxonomic relevance almost immediately after publication, the scientific names established within such catalogs retain their nomenclatural relevance indefinitely. Ultimately, this is why the career-long labors of Sherborn have retained their value well beyond his own life, up until today and continuing indefinitely into the future.

## The more things change, the more they stay the same

**Figure 1.**
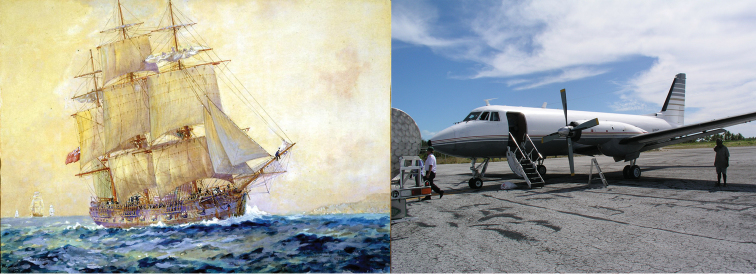
In centuries past, months-long journeys aboard sailing ships were required for taxonomists to reach their destinations (left, Thomas Whitcombe). Today, almost any part of the world can be reached aboard modern aircraft (right, R. L. Pyle).

**Figure 2.**
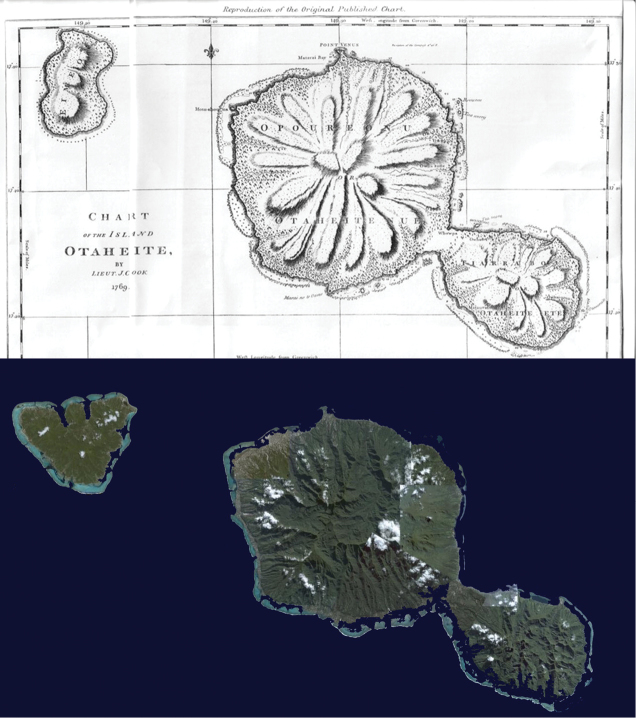
Early taxonomists had only crude maps to plot the locations of their specimens; in this case the French Polynesian islands of Tahiti and Moorea (top, from Prévost D’Exiles 1746–1789). Today, highly accurate maps and satellite imagery can pinpoint particular locations within a few meters (bottom, Landsat).

**Figure 3.**
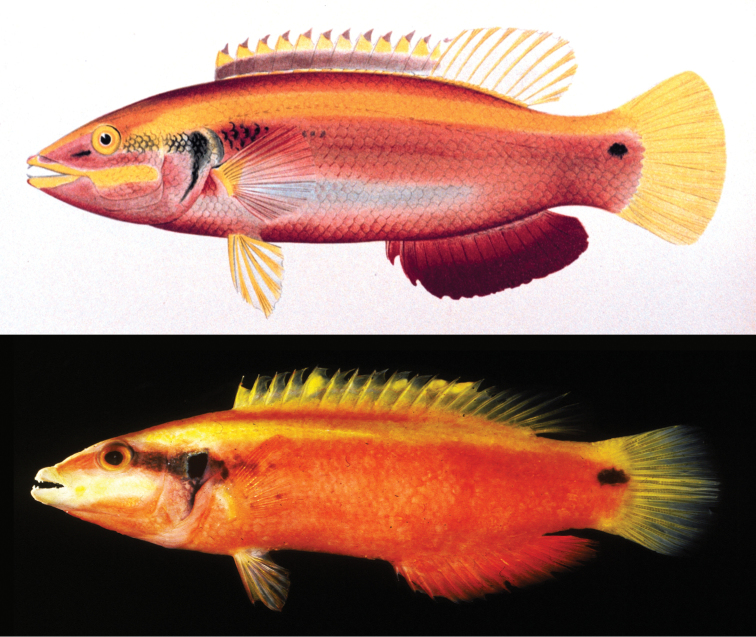
Highly trained artisans once labored to produce detailed hand-painted illustrations of specimens (top, from Jordan and Evermann 1903). Modern digital cameras can generate far more accurate and detailed images almost instantly and with minimal skill (bottom, R. L. Pyle). Both images depict *Bodianus sanguineus* Jordan and Evermann 1903.

**Figure 4.**
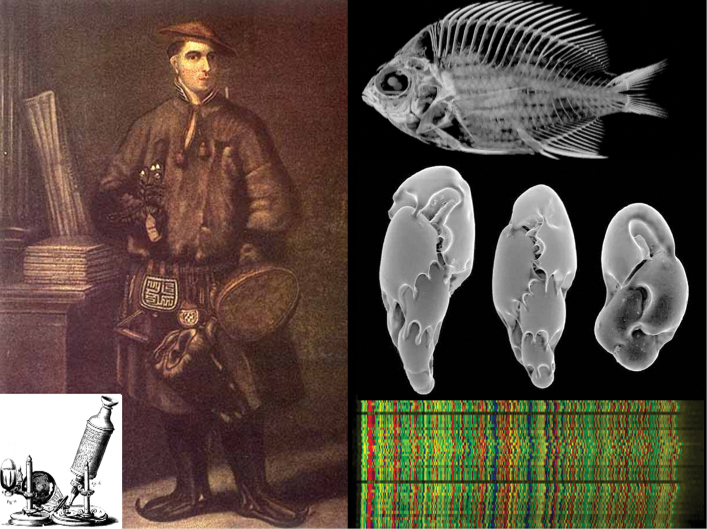
Carl Linnaeus used candle-lit microscopes with primitive optics to examine his specimens (left, H. Kingsbury). Modern technology allows us to generate high-resolution 3D CT scans of the internal structures of specimens without displacing a single scale (right top, Digimorph; *Chromis abyssus*), capture crisp images of tiny organisms through electron microscopy (right middle, NOAA; single-celled foraminifera), and read DNA sequences (right bottom, BOLD, unspecified taxon).

**Figure 5.**
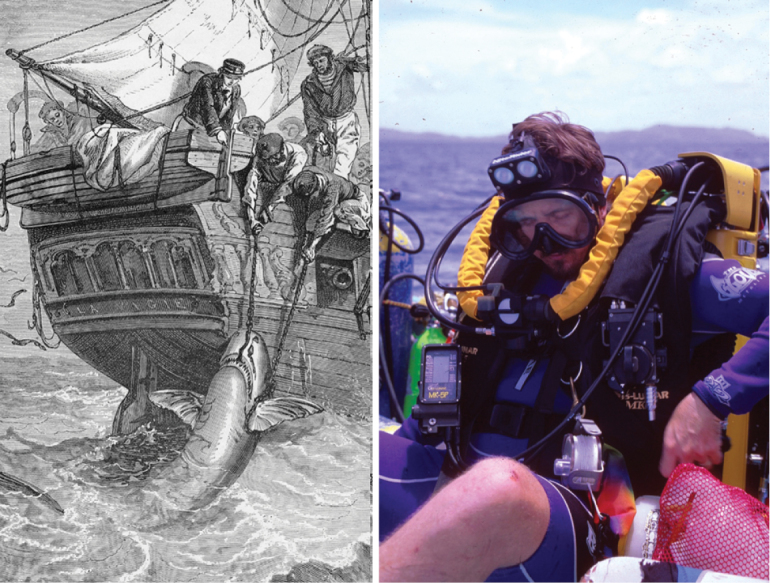
Methods of collecting specimens from the field have advanced from earlier eras (left, from C.Delon, 1889) to modern high-tech equipment of today (right, Ken Corben).

The system of scientific nomenclature is not the only aspect of the taxonomic enterprise that has remained relatively constant over the centuries. Certainly there have been some improvements to the way taxonomists do their jobs. For example, it once required months to journey across the seas aboard sailing ships, whereas now almost any part of the world can be reached within a few hours aboard modern jet airplanes (Figure 1). Early naturalists had to rely on crude maps drawn by sailors to figure out where their specimens were collected, whereas the Global Positioning System (GPS) and digital mapping tools such as Geographic Information Systems (GIS) and Google Earth allow modern taxonomists to pinpoint the collection location for a specimen within a meter or so (Figure 2). A century ago, highly skilled illustrators painstakingly created colorful works of art by hand, based on direct observations and descriptions of the color and form of living organisms, whereas modern digital imaging technology allows us to generate extremely high quality photographs of living and freshly prepared specimens in an instant (Figure 3). To examine his specimens, Linnaeus used primitive candle-lit microscopes with hand-ground optics, whereas today we can generate high-resolution three-dimensional images of the internal and external structures of organisms using 3D photogrammetry and CT scanning without even dissecting them, create crisp images of tiny structures using electron microscopy, and read the very code of life by sequencing DNA (Figure 4). Finally, the technology we use to access the environments in which organisms live has changed dramatically from centuries past (Figure 5).

**Figure 6.**
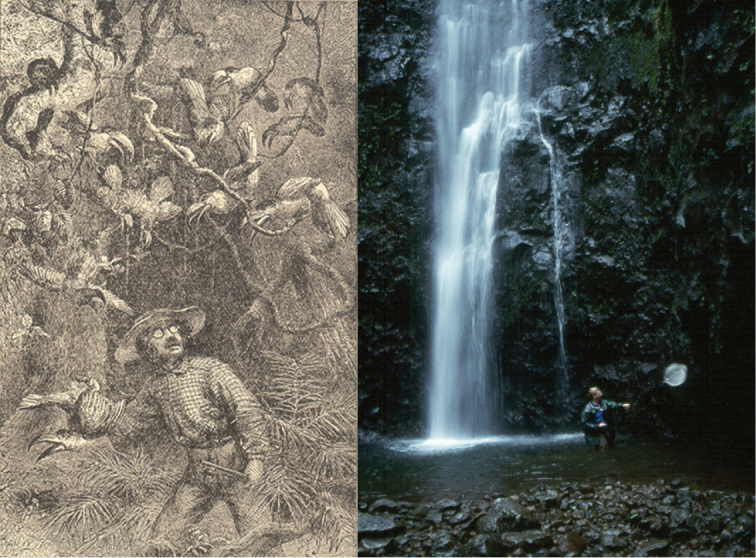
Despite many technological advancements in the tools of the taxonomic trade, the fundamental paradigm for the taxonomic enterprise remains almost unchanged from centuries ago (left, from Bates 1863; right Bishop Museum).

However, despite these important technological advancements in the tools of the trade for taxonomy, the fundamental process remains the same today as it was centuries ago: impassioned naturalists seek financial support from governments and private entities to travel the globe to discover new species of organisms; they take detailed notes and acquire specimens, which they carefully transport back to Museums; they create color images, dissect, poke, prod, count, and measure their biological treasures; they write detailed descriptions that are printed on paper in books and periodicals. These fundamental steps in the taxonomic process, while aided by advanced technology, have remained fundamentally unchanged since the time of Linnaeus (Figure 6).

## A revolution in information technology

There is one aspect of technological change that has been truly revolutionary, which is the means by which taxonomists manage and communicate the information of their trade. The rapid advancement in recent decades of computer database management software, and of information dissemination via the Internet, have both dramatically improved the potential for streamlining the entire taxonomic process. Less than two decades ago, graduate students in taxonomy spent untold hours in libraries, scouring through pages and pages of paper documents to find original descriptions and key taxonomic revisions. Today, with a few searches on Google and with the extremely useful Biodiversity Heritage Library, many original sources are only a few mouse clicks away. And the ease of access is not limited to digitized literature; specimens, images, and vast amounts of biological information are freely available through the Internet. One of the last remaining barriers to information availability – the pay-walls behind which many newly published research hides – is gradually eroding through an increasing demand for open-access models of publication.

**Figure 7.**
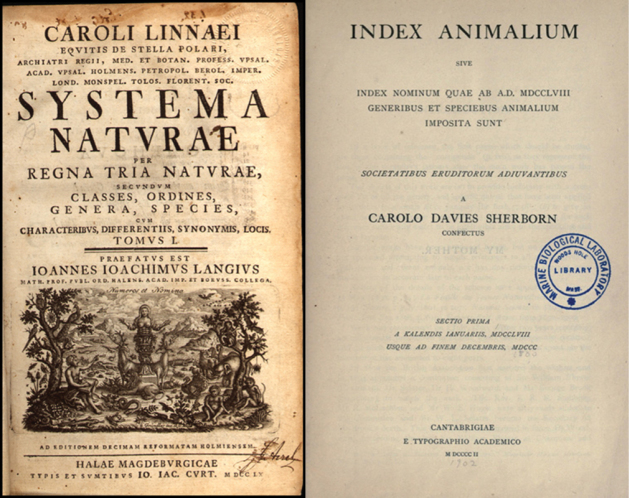
At the start of modern zoological nomenclature, Linnaeus’ tenth edition of *Systema Naturae* contained almost 4,400 species-group names (left). By 1850, the number of species names for animals had reached nearly 430,000 – an increase of two orders of magnitude.

This revolution in digital information technology is extremely fortunate, given the rate at which species have been (and continue to be) described. In 1758, the tenth edition of Linnaeus’ *Systema Naturae* contained nearly 4,400 species-group names. At the time, this compilation represented the entire catalog of all known animals. In the century that followed, the number of scientific names for species had increased by two orders of magnitude, as represented in the volumes of Sherborn’s *Index Animalium* (Figure 7). Today, the online edition of the Catalogue of Life includes more than 2.7 million names (representing more than 1.5 million species). Sherborn spent most of his professional career compiling what is effectively one sixth the number of names that likely exist in biology today. Without the electronic information revolution, the Catalogue of Life would be far less complete than it currently is.

As exciting as the electronic information revolution is, however, in the context of taxonomy there is still far more potential than there is reality in terms of harnessing the power of information technology. The vast majority of taxonomic information either remains non-digitized, or is digitized in a form that does not allow direct and easy access. Moreover, much (if not most) of the information that is easily accessed in digital form is not yet seamlessly interconnected. At present, the total biodiversity knowledge-base for all life forms is scattered across an estimated half-billion pages of printed literature, thousands of natural history collections housing billions of specimens, hundreds of thousands of digital databases and websites, and hundreds of millions of DNA sequences. Consumers of this knowledge-base, which includes tens of thousands of taxonomists, hundreds of thousands of biologists, a hundred million citizen scientists, governmental resource managers and policy makers, and ultimately much of the total human population, have not had easy access to this information (Heidorn 2008; IISE 2010; Thessen and Patterson 2011; Fontaine et al. 2012). There are many excellent websites containing valuable information, including nomenclatural, taxonomic, biogeographic, life-history and ecological information about species, not to mention genetic data, images and videos, and countless other data sources. To find all this information – even when it is readily available through the Internet – usually requires multiple web searches and visits to dozens of different online resources.

The next step in the information revolution for biodiversity information involves not just the digitization of content, but will involve the cross-linking and more seamless integration of existing digital resources.

## The global names architecture

In an effort to bring reality closer to potential, a loose affiliation of major taxonomic resources, including the Global Biodiversity Information Facility (GBIF; http://www.gbif.org), the Encyclopedia of Life (EOL; http://eol.org), the former U.S. National Biological Information Infrastructure (NBII), Catalog of Life (CoL; http://www.catalogueoflife.org), the Integrated Taxonomic Information System (ITIS; http://www.itis.gov), the International Plant Names Index (IPNI; http://ipni.org), the International Commission on Zoological Nomenclature (ICZN; http://iczn.org), Index Fungorum (IF; http://www.indexfungorum.org), and many others have been crafting a “Global Names Architecture” (GNA). The intention of the GNA is not to replace any of the existing taxonomic or other biodiversity data initiatives, but rather to serve as a dynamic suite of web services and two primary indexes (GNI and GNUB, described below) that interconnect existing data systems in a way that streamlines the entire taxonomic enterprise: from gathering specimens in the field, to publication of new taxa and related data.

**Figure 8.**
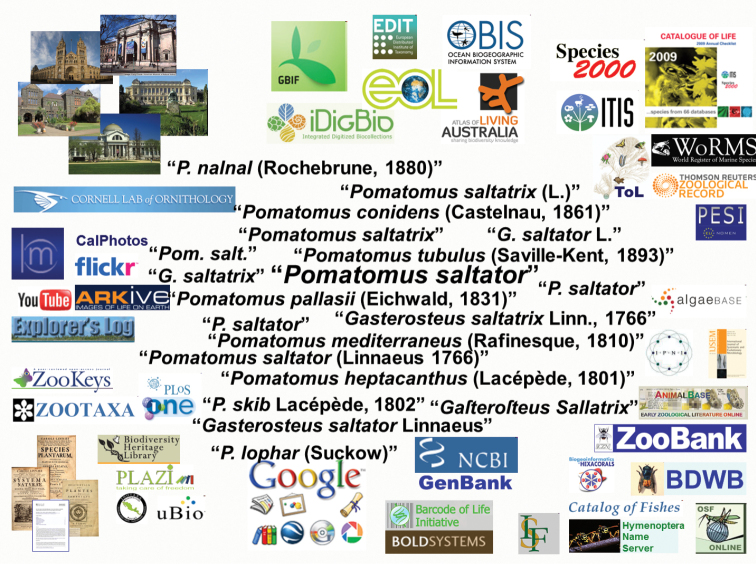
The icons around the periphery represent examples of where biological data tagged with scientific names currently exist. The cluster of names in the center represent examples of distinct text-strings that have been used to represent the same species within different data sources.

The basic premise behind the GNA is that scientific names of organisms represent the key to integrating disconnected biological data, to allow efficient and effective coordination between biological research and exploration activities, and broader understanding and management of biodiversity (Patterson et al. 2010). Throughout the vast global biological knowledge base – including natural history collections, historical and modern literature, observational databases, multimedia (image and video) resources, genetic data repositories, nomenclators, taxonomic catalogs, data aggregators, and major Internet search engines – the majority of data are given taxonomic context through simple text-string scientific names (Figure 8).

Unfortunately, sources of imprecision and ambiguity severely limit the use of these text-string names for cross-linking digital data content. For example, there are approximately two million scientifically described species (Chapman 2009; Trontelj and Fišer 2009; Mora et al. 2011); yet the Global Names Index (GNI) – the component of the GNA that indexes text-string scientific names – already contains nearly 20 million distinct name-strings (and this index is far from complete). The more than ten-fold discrepancy between species and text-string names results from several factors, including synonymy (multiple names for the same species), alternate nomenclatural combinations (the same species epithet combined with different generic and subgeneric names, and applied at different ranks), alternate spellings (orthographic variations, misspellings, abbreviations, etc.), and inconsistent formatting of names (e.g., with and without qualifiers, alternate formats for authorships and/or year, etc.). Additional confusion results from homonymy (the same name assigned to different species).While text-string names are generally easy to interpret and disambiguate by a human, they represent a substantial barrier to electronic cross-linking of data.

To overcome the limitations of text-string scientific names, the GNA includes a core component called the Global Names Usage Bank (GNUB). GNUB is a highly normalized database system, the primary purpose of which is to index and assign persistent globally unique identifiers (GUIDs) to Agents, References, and Taxon Name Usage (TNU) instances (among other relevant data objects). Agents are people and organizations, and in the context of GNUB mostly represent Authors of References. References include all published literature, as well as many forms of unpublished documentation (e.g., unpublished reports and manuscripts, specimen labels, herbarium sheets, field notes, etc.). Any static documentation source can be a Reference in the GNUB architecture. A TNU is any usage or treatment of a scientific name within a Reference. TNUs are the foundation for all Code-governed nomenclatural acts, taxon concept definitions, taxonomic treatments, synonymies and classifications, and any other forms of taxonomic assertions. The subset of TNUs that represent the establishment of new scientific names (i.e., original descriptions) are called “Protonyms” (Pyle 2004). Every scientific name (at every rank) has one Protonym TNU, and all subsequent TNUs refer back to the Protonym. For example, the fish genus *Gasterosteus* was described by Linnaeus (1758), so the TNU for that name in that Reference is the Protonym. Linnaeus (1766) also treated the genus *Gasterosteus*, and that TNU links back to the Protonym TNU in Linnaeus (1758). Protonyms apply to names at all ranks. For example, Linnaeus 1766 established the new species *Gasterosteus saltatrix*. Whereas the TNU for *Gasterosteus* within this publication is not the Protonym for that genus, the TNU for the species epithet *saltatrix* within Linnaeus 1766 is the Protonym for that species-group name (because this publication is the original description of the species, but not the genus).

The core elements of a TNU include the following items (see Table 1; not all elements are required for all TNUs):

1. A unique and persistent identifier for the TNU itself;
2. A link to the Reference (including page, if applicable) in which the TNU appears;
3. A recursive link to the Protonym-TNU for the name represented by the TNU;
4. An indication of the taxonomic rank at which a name was treated (e.g., “genus” for the TNU for *Gasterosteus* within Linnaeus 1766, and “species” for the TNU for *saltatrix* within Linnaeus 1766);
5. The exact spelling (as best as can be represented using UTF-8 encoding) of the name as used within the Reference (e.g., Regan 1909 spelled the genus *Gasterosteus* as “*Gastrosteus*”, and Günther 1860 spelled the species *saltatrix* as “*saltator*”);
6. A link to the TNU (within the same Reference) representing the immediate parent taxon (e.g., the Protonym TNU for the species *saltatrix* within Linnaeus 1766, would link to the non-Protonym TNU for the genus *Gasterosteus* as treated by Linnaeus 1766);

**Table 1.** Examples of Taxon Name Usage instances (TNUs). Records representing Protonyms are highlighted in yellow. Records representing treatments of a name as a synonym is highlighted in grey. Ellipses (…) in the Parent column represent links to higher-rank TNUs not included in the sample below. This table is highly simplified and does not represent the actual GNUB data model.

TNUID	Reference	Protonym	Rank	Spelling	Parent	Valid	Representative Usage
**1**	**Linnaeus 1758:295**	**1**	**Genus**	***Gasterosteus***	…	**1**	**Protonym of genus *Gasterosteus***
2	Linnaeus 1766:489	1	Genus	*Gasterosteus*	…	2	Subsequent usage of *Gasterosteus*
**3**	**Linnaeus 1766:491**	**3**	**Species**	***Saltatrix***	**2**	**3**	**Protonym of species *Gasterosteus saltatrix***
4	Schöpf 1788:167	1	Genus	*Gaſteroſteus*	…	4	Subsequent usage & variant of *Gasterosteus*
5	Schöpf 1788:168	3	Species	*Sallatrix*	4	5	Subsequent usage & variant of *Gasterosteus saltatrix*
**6**	**Lacépède 1802:435**	**6**	**Genus**	***Pomatomus***	…	**6**	**Protonym of genus *Pomatomus***
**7**	**Lacépède 1802:436**	**7**	**Species**	***Skib***	**6**	**7**	**Protonym of species *Pomatomus skib***
**8**	**Cuvier 1816:346**	**8**	**Genus**	***Temnodon***	…	**8**	**Protonym of genus *Temnodon***
9	Günther 1860:479	8	Genus	*Temnodon*	…	9	Subsequent usage of *Temnodon*
10	Günther 1860:479	3	Species	*Saltator*	9	10	Subsequent usage & variant of *Gasterosteus saltatrix*
11	Günther 1860:479	7	Species	*Skib*	-	10	Synonym treatment of *Pomatomus skib*
12	Bean 1903:445	6	Genus	*Pomatomus*	…	12	Subsequent usage of *Pomatomus*
13	Bean 1903:445	3	Species	Saltatrix	12	13	Subsequent usage & combination of *Gasterosteus saltatrix*

In cases where a name is treated as a junior synonym of another name, a link to the TNU (within the same Reference) representing the senior synonym as asserted by the indicated Reference for this junior synonym (e.g., Günther 1860 treated the name *Pomatomus skib* (Lacepède 1802) as a junior synonym of *Temnodon saltator* (Linnaeus 1766), so the TNU for the Günther 1860 treatment of *skib* links to the TNU representing the Günther 1860 treatment of *saltator* [=*saltatrix* Linnaeus 1766]).

By building an index of all TNUs across historical literature (starting with the Protonym TNU for each name), GNUB data services can efficiently perform powerful analyses and transformations of taxon names across different spellings, synonymies and classifications. For example, the species *Gasterosteus saltatrix* Linnaeus, 1766, has also been spelled *Sallatrix* in at least one Reference, spelled *saltator* in at least 16 References, and the species epithet (by whichever spelling) has been variously combined with the genus names *Pomatomus* Lacépède, 1702, *Temnodon* Cuvier, 1816 and *Cheilodipterus* Lacépède, 1801. Moreover, the GNUB index also records the fact that at least twelve other species have been treated as a junior synonyms of *saltatrix*, and these species have been variously combined with at least ten different genus names. Thus, through GNUB we can see that the species originally established by Linnaeus 1766 as *Gasterosteus saltatrix* has been variously referred to in literature by at least 28 different text-string scientific names (inclusive of both homotypic and heterotypic synonyms, suite of GNUB and GNI services, content in otherwise disconnected datasets can be cross-linked despite heterogeneous taxon names.

## A successful proof of concept

Largely through support from two separate NSF grants (DBI-1062441; DBI- 0956415), the GNA has been developed into a highly successful proof of concept. The most visible representation is the ZooBank registry (http://zoobank.org). ZooBank was first proposed as an official online nomenclatural registry for zoology, under the auspices of the International Commission for Zoological Nomenclature (ICZN) by Polaszek et al. 2005. It was first launched as an early prototype on 1 January 2008 to commemorate the 250^th^ anniversary of the official start of all zoological nomenclature (Pyle et al. 2008; Pyle and Michel 2008; Pyle and Michel 2010; Rosenberg et al. 2012). ZooBank was later reconceived as a service operating on top of GNUB (Pyle and Michel 2009), and the new GNUB-based ZooBank was publicly launched on September 4, 2012, coinciding with the amendment to the ICZN Code supporting electronic publication (ICZN 2012a; 2012b). The amended Code requires all electronically published works in Zoology be registered in ZooBank, thus representing the first mandatory electronic/online registration requirement for any major Code of Nomenclature (the bacteriological Code includes a paper-based registration system [Tindall 2009], and the Code for algae, fungi and plants includes a registration system for fungal names that went into effect on 1 January 2013).

Prior to 2012, ZooBank registrations grew steadily from approximately 100 registrations per month in 2008-2010, to approximately 500 registrations per month in 2011–2012. After the new GNUB-based implementation of ZooBank was launched in September 2012, registrations increased almost ***ten-fold***, to nearly 5,000 per month. The vast majority of these regiwstrations are prospective – that is, for works and names that are newly established. Retrospective content for ZooBank will be added through the bulk importation of existing databases, and through harvesting protonyms from BHL and other sources. Commensurate with the rise in registrations has been an increase in the ZooBank user-base. From 2008–2012, the ZooBank user base grew steadily to a little over 100 active users. In less than a year since the GNUB-based ZooBank was launched, the user base has also grown nearly ***ten-fold***, to over 1,000 users (and it continues to grow). As successful as the new GNUB-based ZooBank has been, it is important to emphasize that ZooBank is only one example of a service that GNUB can facilitate. In addition to ZooBank as a model for GNUB-based registration systems in other nomenclatural domains, there are many other services that GNUB can facilitate.

Whereas name-usages within static References are indexed directly as TNUs, these are mapped to records in external and/or dynamic data sources through a simple identifier cross-link feature in GNUB. This feature, which currently includes nearly half a million links from records in more than 200 external databases to over 320,000 GNUB records, enables much more than simply linking GNUB records to external databases; specifically, it allows external databases to be linked to ***each other***.

For example, GNUB includes links to over 111,000 registered names in ZooBank, nearly 140,000 records (taxonomic serial numbers) in ITIS, and nearly 70,000 genus-group and species-group name records in the Catalog of Fishes (CoF). Besides allowing these three datasets to link directly to GNUB (and vice-versa), the Identifier cross-link service also enables direct cross-links between each of these otherwise disconnected datasets (in this case, 67,000 linked records between ZooBank and CoF; 26,555 linked records between ZooBank and ITIS, and 26,467 linked records between ITIS and CoF). Because of this cross-linking feature, new names registered in ZooBank could be presented to ITIS and CoF for inclusion in their databases, and corrections made to errors in CoF could be propagated to both ZooBank and ITIS. By establishing cross-links between equivalent records in different database systems, we not only expand the ability for end-users to directly access records in the different systems, but we also create novel opportunities for proactive collaboration between different systems with overlapping content. While other systems include support for similar features (e.g., the EoL “Partner Links”, and the NCBI Taxonomy “LinkOut”), the GNA provides a single shared platform for all cross-links, such that anytime a record is indexed in GNUB, it is automatically cross-linked to all other data systems that are indexed in GNUB.

This cross-linking service is not limited to taxon names. For example, GNUB includes links to over 3,300 journals registered in ZooBank and over 3,200 journals scanned in the Biodiversity Heritage Library (BHL). Through the BHL “OpenURL” service, over 50,000 ZooBank species pages (as well as nearly 100,000 other TNU records) now have direct access to the corresponding page image in BHL. Likewise, because GNUB is linked to over 34,000 authors in the Authors of Plant Names (APN) directory, and nearly 21,000 authors registered in ZooBank, we can compare authorship trends in both domains (e.g., fewer than 1% of all authors have published new scientific names for both plants and animals).

This same cross-linking service applies to records in more than 200 different external databases (not all databases have been fully indexed yet). As such, GNUB can serve as a universal hub to cross-link records (not only Authors, References, and TNUs/Protonyms; but virtually any other data object as well), which will facilitate collaboration and data exchange (as in the names-linking example), enhance web services to infer and establish other links (as in the BHL page example), and to allow analysis of patterns that had not previously been possible (e.g., patterns of authorship over time, such as those as used by Costello et al. 2012). This cross-linking service developed for GNUB represents an important step towards empowering the collective utility of biological datasets on a global scale.

Several other services and APIs were developed for searching, dereferencing, editing, and inserting Agents, References, and TNUs (particularly Protonyms), with a variety of output formats (e.g., HTML, JSON). The most recent service is called “GNIE” (originally an acronym for “Global Names Index Export”, and retained despite its expanded utility), which accepts an identifier for a Protonym and returns a set of all scientific names indexed in GNUB that have been used to represent the same taxon, including all homotypic synonyms (spelling variants, alternate genus combinations, etc.) and heterotypic synonyms (names that have been treated as either junior or senior synonyms of the indicated Protonym). Documentation for all of these services is included on the ZooBank API page (http://zoobank.org/Api).

**Figure 9.**
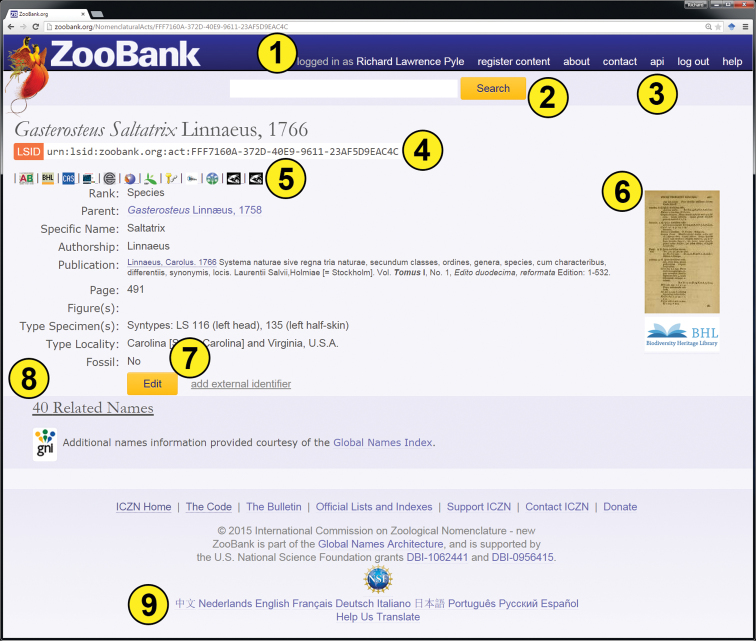
An example ZooBank page, illustrating several GNUB services: **1** user authentication **2** “fuzzy” searching of GNUB content **3** APIs and services **4** ZooBank registration **5** External Identifier cross-linking **6** BHL page linking **7** record editing capabilities **8** similar/related name discovery (via GNI’s name searching service); and **9** multi-lingual support. Not shown are services to manage user accounts, de-duplicate records, prototype reconciliation tools, services for journal publishers, and visualization tools for author publication history and other statistics.

Although these services were used extensively in the development of ZooBank (Figure 9), funding from NSF supported the development and implementation of these and other as-yet undocumented services in various stages of development and testing on two other database systems as well: Bishop Museum natural science databases (http://nsdb.bishopmuseum.org/), and the “Explorer’s Log” (http://www.explorers-log.com/). Bishop Museum manages several major specimen and occurrence databases (Plants, Insects, Birds, Mammals, Amphibians and Reptiles, Fishes, Marine Invertebrates, Mollusks, and Pacific Center for Molecular Biodiversity, as well as several regional checklist databases), and we are currently in the process of building support for GNUB as the taxonomic authority against which Bishop Museum specimen databases are indexed. The “Explorer’s Log” (http://www.explorers-log.com) is a feature-rich suite of web-based applications designed to support field-based data collection for organismal occurrence records (including specimens and associated tissue samples, multi-media documentation such as images, videos, audio recordings and telemetry data, visual observations, and literature-based occurrence records) and associated data. This system has now completely replaced its internal taxonomy tables and utilizes GNUB services to assign taxonomic identifications to organism occurrences. The purpose of developing these prototype services was to demonstrate the ability for external data management systems to utilize GNUB data and web services directly to support broader biological datasets, without the need to re-invent a custom taxonomic authority system (as is currently done for most biological data management systems).

In addition to these services designed primarily to support external systems, several services designed for internal GNUB use were also developed. These include a user/login account management system, a robust record de-duplication resolution system, a prototype data reconciliation tool (currently optimized for Agents and journal titles) used for bulk data imports, multi-lingual support, a tool for visualizing the publication timeline history for authors, a suite of database statistics visualization services, and services to facilitate data contribution and management by publishers and editors of journals.

### An integrative infrastructure for biodiversity data

Unlike most existing biodiversity data initiatives, the components of the GNA are not primarily intended to provide novel information; rather, the GNA includes an index of core facts (and associated data services) that are shared across all of biology. Nothing in the GNA is original or novel content; it merely represents a structured way of organizing information to facilitate broader data integration among other databases that do contain original information. Thus, the GNA does not compete with other data resources; but rather serves as a core infrastructure for cross-linking (and thereby empowering) other biological data sources.

Although the GNA is primarily intended to provide a cross-linking service between existing databases, the data model is sufficiently robust and complete that it can fulfill the primary needs of representing nomenclature, taxonomy and classification for groups that are not otherwise represented by existing databases. Thus, while its primary function is to integrate biodiversity data across multiple disparate systems, the GNA is capable of filling the gaps in taxonomic coverage for groups of organisms not already well-represented in the broader biodiversity data landscape.

## Salvaging the global biodiversity library

Biodiversity is Earth’s greatest Library, representing the culmination of information that has been written and re-written, edited and re-edited, over the past four billion years. We are like Kindergartners running through the Library of Congress: surrounded by vast amounts of incredibly valuable information – the genomic equivalents of the works of Homer, Shakespeare, and blueprints for a nuclear power plant and 95% efficient conversion of sunlight energy to stored chemical energy, but we are currently only able to interpret this information at the equivalent of “See Spot Run”. Someday soon (within the next few decades) we will have the ability to truly understand the information in the Biodiversity Library. As we face the 6^th^ Great Extinction event, we recognize that the Biodiversity Library is burning, so the information will be gone before we have a chance to understand its true value. Whenever a species goes extinct, it’s like burning the last copy of a book. Taxonomists are the Librarians, and have perhaps the most important job of all: building the digital equivalent of the “card catalog” for the Biodiversity Library.

This audacious task was begun in the 1750s by Carl Linnaeus, and was dramatically extended by Charles Davies Sherborn 150 years later. With the advent of modern electronic information management, we are poised to achieve the vision of these two pillars of science; but we are in a race against the destruction of what we seek to document. We are the first generation in human history to understand our own impact to biodiversity, and we are very likely the last generation in a position to do anything about it. The Global Biodiversity Library is burning, and we must tirelessly continue to document the richness of form and function in nature before it is lost forever.

## Conclusions

Throughout most of the history of modern taxonomy and nomenclature, the basic tasks performed by taxonomists have remained remarkably unchanged. Technology has allowed some improvements, with modern electronic information dissemination representing the most significant advancement. At this point in history, biodiversity data are being digitized at an impressive rate, but in most cases the data remain in “silos”, with limited interconnectivity. As such, the accumulated digitized data cannot be used to its full potential. The most effective way to integrate disparate biodiversity datasets is through scientific names, but for many reasons, text-string names alone are not effective for this purpose. The Global Names Architecture (GNA) has been developed to provide core indexes and cross-linking services, to help leverage otherwise disparate biodiversity data. ZooBank, the official online registry of zoological nomenclature, represents only one example of how the GNA can improve interconnectivity among biodiversity data. Going forward, the priority should be to continue digitizing data, and to develop robust cross-links among existing biodiversity datasets. Global biodiversity is precious – perhaps Earth’s most valuable resource – yet we have only begun to catalog its contents. With global climate change and accelerating rates of extinction, it is more important than ever to extend the work of Charles Davies Sherborn to apply to all known and yet-to-be-discovered taxa.

Sherborn himself seemed to understand the challenges of his task, many of which remain true today. In the Epilogue of *Index Animalium*, (Section 2, Part 29, pp. vi-vii), he wrote:

*Now my work is finished, it may well be to glance at the difficulties met with during compilation… This want of every book and every edition has been a serious hindrance and loss of time to me while working for over forty years in the British Museum (Natural History) and though I have acquired over a thousand volumes for the libraries, gaps still remain to be filled… On the whole one has met with a generous response, but the amused smile, real apathy, or the remark ‘we have no money’ … have been encountered*…

He was also acutely aware of the nature of evolving technology:

*And now that rotography has superseded photography as regards cost, a rare tract can be reproduced in a few hours and placed on its proper shelf in any Library for a few shillings*.

But most important of all, Sherborn understood the grandeur of his quest, and knew full well that it was far greater than his own personal contributions:

*In conclusion I may add that the whole of my papers, Books of Reference and apparatus will remain at the Museum for my continuator and I trust that arrangements will be made for the permanent indexing of even current literature as the only true method of economizing the time of the working zoologist*.

It is our responsibility as modern biologists to harness the power of new technology to continue is this all-important task of documenting biodiversity.
